# FAHD1-mediated pyruvate metabolism in hepatocellular carcinoma: Multi-omics and causal genetic evidence

**DOI:** 10.1016/j.xhgg.2025.100494

**Published:** 2025-08-14

**Authors:** Jin Huang, Shijie Liang, Jiamin Sun, Huaping Chen

**Affiliations:** 1 Key Laboratory of Clinical Laboratory Medicine of Guangxi Department of Education, Department of Clinical Laboratory, the First Affiliated Hospital of Guangxi Medical University, Nanning, Guangxi, China

**Keywords:** single-cell RNA sequencing, spatial transcriptomics, Mendelian randomization, hepatocellular carcinoma, pyruvate metabolism, FAHD1, AlphaFold2, molecular docking, immune evasion, tumor microenvironment

## Abstract

Hepatocellular carcinoma (HCC) progression is driven by metabolic reprogramming in the tumor microenvironment (TME), yet the causal regulators of pyruvate metabolism and their spatial interplay remain elusive. Here, we integrate single-cell transcriptomics, spatial mapping, and genetic causal inference to identify a pyruvate-hyperactive epithelial subpopulation (PyHighEpi) in HCC, characterized by enhanced stemness, proliferation, and metastatic traits. Spatial analyses reveal metabolic zonation, with pyruvate activity concentrated in tumor cores and associated with aggressive clones. Summary data-based Mendelian randomization identifies fumarylacetoacetate hydrolase domain containing 1 (FAHD1) as a potential causal driver, with its expression associated with a poor prognosis. FAHD1+epi cells interact with cancer-associated fibroblasts through ITGB2-mediated interactions, facilitating the formation of a transforming growth factor-β/vascular endothelial growth factor-enriched niche that promotes immune evasion. Clinically, FAHD1 overexpression correlated with poor prognosis, validated through functional assays showing its knockdown suppressed proliferation, invasion, and migration in HCC models. An FAHD1-derived risk score robustly stratifies patient prognosis and predicts responsiveness to immunotherapy, while molecular docking highlighted tivozanib as a potential FAHD1-targeting agent.

## Teaser

Hepatocellular carcinoma (HCC) progression is linked to disrupted metabolism. We identified FAHD1 as a key regulator of pyruvate metabolism in HCC through multi-omics analysis and experimental validation. FAHD1 overexpression correlates with poor prognosis and shapes the immunosuppressive tumor microenvironment, revealing its potential as a therapeutic target to improve HCC outcomes.

## Introduction

Hepatocellular carcinoma (HCC) remains a leading cause of cancer-related mortality, with persistently poor prognosis despite multimodal therapeutic advances.^1^ This therapeutic recalcitrance stems from the dynamic reciprocity between neoplastic hepatocytes and their tumor microenvironment (TME), where metabolic cross-talk sustains immune privilege and metastatic dissemination.^2^^,^^3^ Among the hallmarks of TME remodeling, metabolic reprogramming has emerged as a critical driver of tumor adaptation, enabling cancer cells to thrive under hostile conditions and resist therapeutic interventions.^4^^,^^5^ While Weinbergian paradigms dominate HCC metabolism research, the spatiotemporal regulation of pyruvate metabolic nodes across evolving tumor ecosystems remains uncharted.

Pyruvate metabolism, functioning as a critical metabolic nexus coordinating oxidative phosphorylation (OXPHOS) and glycolysis, extends beyond its role as a metabolic intermediate to actively shape tumor dynamics, promoting invasion and migration across diverse malignancies.^6^^,^^7^ In breast cancer, pyruvate carboxylase (PC)-mediated anaplerotic entry into the tricarboxylic acid (TCA) cycle promotes an invasive phenotype by enhancing cell motility.^8^ Conversely, disruption of PC activity reduces reduced and oxidized forms of the coenzyme nicotinamide adenine dinucleotide phosphate and reduced and oxidized glutathione ratios, impairing reactive oxygen species (ROS) scavenging and increasing oxidative stress, thereby facilitating the survival of circulating tumor cells.^9^ While in HCC, dimeric pyruvate kinase M2 stabilization by HSP90 drives glycolytic addiction and apoptotic resistance.^10^ Moreover, the E1α subunit of pyruvate dehydrogenase regulates nuclear factor κB (NF-κB) signaling, linking mitochondrial metabolism to immune evasion.^11^ These findings underscore pyruvate metabolism as more than a passive by-product of oncogenesis—it is an active driver of tumor evolution and therapeutic resistance.

**Figure 1:**
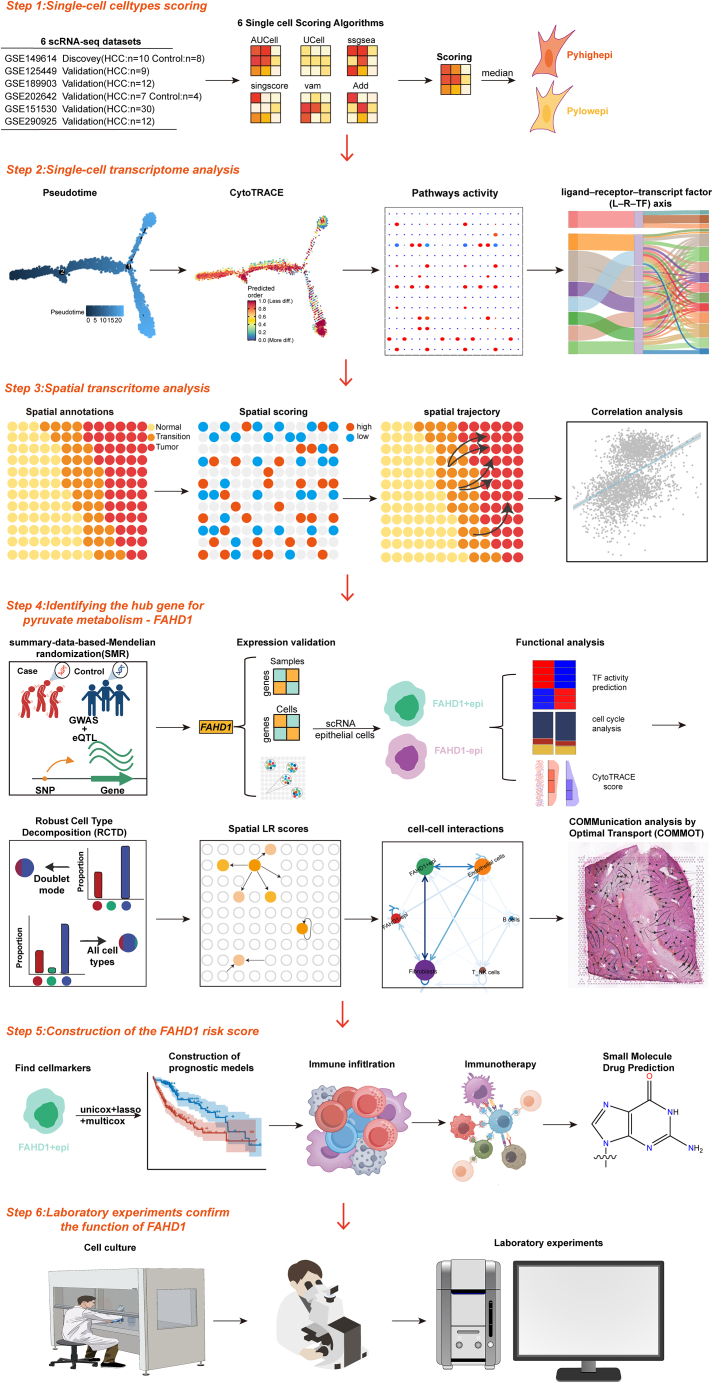
The workflow of the study

Here, we developed an integrative framework that combines single-cell RNA sequencing (scRNA-seq) with spatial transcriptomics (ST) and summary data-based Mendelian randomization (SMR) to elucidate the role of pyruvate metabolism in HCC. Through this systematic approach, we mechanistically identified fumarylacetoacetate hydrolase domain containing 1 (FAHD1) as a master regulator driving HCC progression. Then, we identified it as a key driver of HCC progression through causal genetic inference, linking its expression to poor prognosis and immune-evasive niche formation. The functional role of FAHD-1 in HCC was validated through clinical specimen analysis and *in vitro* functional assays involving the knockdown of FAHD1. A schematic overview of the analytical workflow is presented in Figure 1. Our study proposes a novel strategy for developing therapies that target pyruvate metabolism.

## Material and methods

### Source of raw data

The study integrated multi-omics data from the following sources: scRNA-seq datasets (GEO: GSE149614,^12^ GEO: GSE125449,^13^ GEO: GSE189903,^14^ GEO: GSE202642,^15^ GEO: GSE151530,^16^ and GEO: GSE290925), and ST data (CNCB: HRA00043) were obtained from the Gene Expression Omnibus (GEO) and the China National Center for Bioinformation (CNCB), respectively. Bulk transcriptomics data were drawn from the cohorts of The Cancer Genome Atlas-Liver Hepatocellular Carcinoma Collection (TCGA:LIHC) and International Cancer Genome Consortium (ICGC:LIRI-JP). For SMR analysis, we utilized *cis*-expression quantitative trait loci (*cis*-eQTL) data from hepatic tissue provided by the Yang lab^17^ and HCC genome-wide association study (GWAS) summary statistics from the FinnGen_R8 cohort, comprising 648 cases and 259,583 controls.^18^ Pyruvate metabolism-related gene sets were curated from the Molecular Signatures Database (MSigDB).

### scRNA-seq analysis

Pre-processed scRNA-seq data were analyzed using the Seurat package (version 4.4.0) in R (version 4.3.2). After normalization (“LogNormalize” method, scale factor 10,000), 3,000 highly variable genes were selected via the variance-stabilizing transformation method. Batch correction was performed with Harmony (version 1.2.0), followed by principal-component analysis for dimensionality reduction, with the optimal principal components selected via ElbowPlot. A cell-cell interaction network was constructed using the FindNeighbors function, and clustering was performed with the FindClusters function. Uniform manifold approximation and projection (UMAP) and t-distributed stochastic neighbor embedding were used for data visualization. Cell types were annotated based on the original dataset and CellMarker database.^19^ Pyruvate metabolism activity was quantified using five computational methods (AUCell, UCell, ssGSEA, singscore, and Vam) implemented in the irGSEA package (version 2.1.5)^20^ alongside Seurat’s AddModuleScore method. Pseudo-time trajectory analysis was performed using the Monocle R package (version 2.30.1).^21^ At the same time, cell-cell communication networks were reconstructed with CellCall (version 1.0.7) by integrating ligand-receptor pairs and transcription factor (TF) activity to establish functional Ligand(L)-Receptor(R)-TF axes.^22^ Cellular stemness and transcriptional regulatory networks were analyzed using CytoTRACE^23^ and DoRothEA,^24^ respectively. CytoTRACE assigned stemness scores ranging from 0 (differentiated) to 1 (stem-like), with higher scores indicating greater stemness (less differentiation) based on per-cell gene counts. DoRothEA predicts TF activity through literature-curated, confidence-ranked (A–E) regulatory networks from the literature and experimental datasets.

### ST data analysis

Raw ST data (HRA000437) were filtered using the Seurat R package to retain spots with more than 300 genes and more than 500 counts, followed by the removal of mitochondrial and ribosomal genes. SCTransform-normalized data underwent PCA-UMAP dimensionality reduction using the first 30 principal components. For spatial cell-cell interaction analysis and pseudo-time trajectory inference, we utilized the stLearn Python package (version 0.4.12).^25^ This tool integrates spatial distance, tissue morphology, and gene expression measurements to provide a comprehensive analysis framework. By leveraging morphological similarities between neighboring ST spots, it normalizes gene expression, mitigating “dropout” noise, a common challenge in scRNA-seq technologies.^26^ Furthermore, stLearn extracts morphological features from H&E-stained images to smooth expression data, improving spatial domain detection and enabling trajectory inference across distinct regions. This SME-normalized strategy effectively captures both spatial and transcriptional relationships among subclusters. Cell-type deconvolution employed the robust cell type decomposition (RCTD) method,^27^ juxtaposing scRNA-seq reference profiles onto spatial coordinates. The direction of spatial signal flow was analyzed using communication analysis by optimal transport (COMMOT).^28^

### SMR analysis

To dissect genotype-phenotype associations mediated by transcriptional regulation, we implemented SMR leveraging liver *cis*-expression quantitative trait loci (eQTLs) as instrumental variables (IVs). This methodology enhances detection power for causal inference compared to conventional MR approaches, particularly when utilizing exposure (eQTL) and outcome (GWAS) datasets derived from independent large-scale cohorts.^29^ All analytical procedures were conducted using SMR software (version 1.3.1) with default parameters via command-line execution. The *cis* region was defined as a 2-MB window flanking the probe in both directions. Genes with at least one *cis*-eQTL showing a P_eQTL_ < 5.0 × 10^−8^ were included. Single-nucleotide polymorphisms (SNPs) with allele frequency differences more significant than 0.2 between the eQTL and GWAS datasets were excluded. Significant SMR signals (*p* < 0.05) were validated using heterogeneity in dependent instruments (HEIDI) tests to exclude linkage disequilibrium confounding (*p* > 0.05).

### Prognostic model development

The TCGA-LIHC cohort (*n* = 367) was used as the training set, while the ICGC datasets (*n* = 239) served as the validation set. Initially, marker genes for the FAHD1+epi subpopulations were identified. Univariate Cox regression analysis was then performed to identify genes significantly associated with overall survival (OS) (*p* < 0.05). Lasso regression was applied, followed by multivariate Cox regression, to further screen for prognostic factors closely related to patient survival. The FAHD1-derived risk score (FRS) for each patient was calculated based on the regression coefficients as follows:

$$Risk\;Score=\sum_{i=1}^{n}\beta i\times \exp\left(i\right)\text{.}$$

Here, *n* represents the number of genes included in the model, while *β* denotes the regression coefficient, and exp(*i*) corresponds to the expression level of each gene. Patients were stratified into two groups based on the median FRS value, and a Kaplan-Meier analysis was performed to assess survival differences between the groups.

### Immunoinfiltration assessment, immunotherapy efficacy prediction, drug prediction, and molecular docking analyses

Immune infiltrations were quantified using the microenvironment cell populations counter (MCP-counter), a gene expression-based computational framework that estimates TME composition by integrating immune and stromal cell signatures.^30^ Immunotherapy responses were evaluated in publicly available cohorts of patients receiving diverse treatments: GSE202069^29^ (anti-programmed cell death protein-1 [PD-1] therapy), GSE109211^30^ (sorafenib), GSE215011^31^ (nivolumab), GSE104580 (transarterial chemoembolization [TACE]), and GSE279750 (anti-PD-ligand 1 [PD-L1] therapy). Drug prediction was performed using the Connectivity Map (CMap, https://clue.io/), a database that identifies potential therapeutic compounds by analyzing correlations between disease-associated and drug-induced gene expression signatures.^32^ We utilized AlphaFold2^33^ to predict the structure of the FAHD1 protein, using its sequence retrieved from the NCBI database, while small-molecule structures were obtained from the PubChem Compound database. Molecular docking studies were then performed using AutoDock Vina 1.5.7.^34^

### Clinical tissue samples

Thirty HCC-tumor/non-tumor pairs of tissues were prospectively collected from treatment-naive patients undergoing curative surgical resection at the First Affiliated Hospital of Guangxi Medical University (June 2024–January 2025). The eligibility criteria were as follows: (1) histologically confirmed HCC and (2) no prior anticancer therapy, including chemotherapy, radiotherapy, targeted agents, ablation, or interventional treatments. The exclusion criteria were as follows: (1) secondary/metastatic tumors, (2) perioperative mortality, and (3) incomplete records. Tissues were stored at −80°C until the total RNA was extracted. The study protocol adhered to the Declaration of Helsinki and was approved by the Institutional Ethics Committee of the First Affiliated Hospital of Guangxi Medical University (no. 2025-E0088), with written informed consent obtained from all participants.

### Cell culture and transfection conditions

Normal human hepatocyte THLE2 cells and HCC cell lines (MHCC97H, PLC/PRF/5, SNU182, Huh-7, and HCC-LM3) were obtained from the Cell Bank of the Chinese Academy of Sciences. Cells were cultured in Dulbecco’s modified Eagle’s medium (Gibco, USA), supplemented with 10% fetal bovine serum (FBS; Gibco), and maintained in a 37°C incubator with 5% CO_2_. For functional studies, FAHD1 knockdown was achieved by transfecting MHCC97H and PLC/PRF/5 cells with short hairpin RNA (shRNA) targeting FAHD1, and using scrambled shRNA as a negative control, and shRNA sequences are provided in Table S1.

### Quantitative real-time PCR and western blotting

Quantitative real-time PCR and western blotting were conducted according to previously outlined protocols.^35^ Primer sequences are provided in Table S1. The FAHD1 antibody (catalog no. 68624-1-Ig) for the western blot was purchased from Proteintech.

### Functional validation experiments

Cell proliferation was quantified via Cell Counting Kit-8 (CCK-8; Lianke Biotechnology, China) by measuring 450 nm absorbance (Thermo Fisher microplate reader) at 0, 24, 48, and 72 h post-seeding (3,500 cells/well). Colony-formation capacity was evaluated by counting ≥50-cell colonies after 10–14 days of culture, fixed with 4% paraformaldehyde (Solarbio, China), and stained with 0.1% crystal violet. Cell invasion was assessed using Matrigel-coated transwell chambers (Corning, USA): 1 × 10^5^ serum-starved cells were placed in the upper chambers, and a 20% FBS chemoattractant was present in the lower chambers. Invaded cells were fixed and stained after 24 h and quantified using ImageJ. For wound healing, confluent monolayers (90%) were scratched with pipette tips, serum deprived, and monitored for 24 h; migration rates were calculated from time-lapse images using ImageJ.

### Statistical analysis

Statistical analyses utilized Python version 0.4.12 (via PyCharm IDE version 3.9) with Pandas version 1.5.3, NumPy version 1.24.4, Scanpy version 1.10.1, stLearn version 0.4.10, and COMMOT version 0.0.3; the analyses also used R version 4.4.0 with Seurat version 4.4.0, harmony version 1.2.0, irGSEA version 2.1.5, Monocle version 2.30.1, CellCall version 1.0.7, CytoTRACE version 0.3.3, DoRothEA version 1.14.1, glmnet version 4.1-8, survival version 3.6-4, survminer version 0.9.4, timeROC version 0.4, IOBR version 0.99.8, and spacexr version 2.2.1. Additional tools included SMR version 1.3.1, AutoDock Vina version 1.5.7, AlphaFold2, SPSS version 23.0, and GraphPad Prism version 8.0. Wilcoxon rank-sum tests compared continuous variables, while Cox regression identified prognostic factors. Spearman’s correlation assessed variable associations. Experimental data are expressed as mean ± SD from ≥3 replicates, with t tests for two-group comparisons and ANOVA for multiple groups. Significance: *p* < 0.05 (∗*p* < 0.05, ∗∗*p* < 0.01, ∗∗∗*p* < 0.001, ∗∗∗∗*p* < 0.0001, ns: not significant).

## Results

### Pyruvate metabolism is increased in tumor epithelial cells

**Figure 2:**
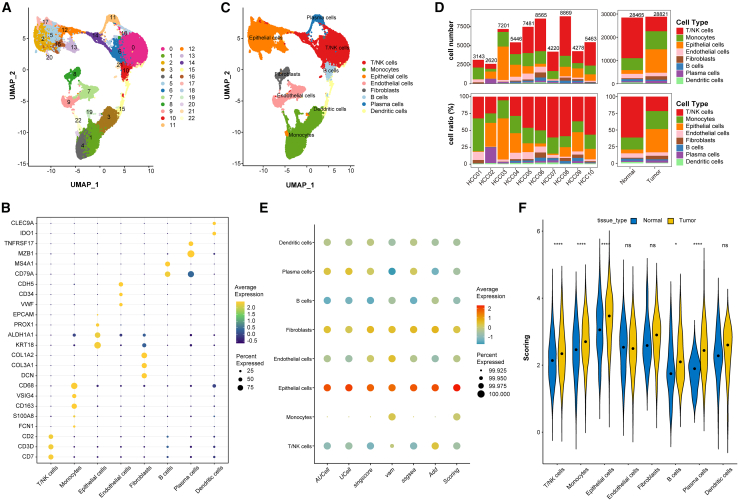
Single-cell transcriptomic profiling reveals elevated pyruvate metabolism in tumor epithelial cells (A) UMAP visualization identifying 57,286 cells across 22 cell clusters. (B) Marker gene expression profiles defining 8 major cell types. (C) Classification of 22 clusters into 8 cell types. (D) Cell number (upper) or ratio (lower) in different tissues. (E) Pyruvate metabolism activity scores across cell types (GSE149614 dataset, multi-method assessment). (F) Violin plots of metabolic scores stratified by tissue type. ∗*p* < 0.05, ∗∗*p* < 0.01, ∗∗∗*p* < 0.001, ∗∗∗∗*p* < 0.0001, ns *p* > 0.05.

Analysis of scRNA-seq data from 10 HCC samples revealed a TME comprising 57,286 cells classified into 22 distinct clusters (Figure 2A). Based on marker gene expression, we classified the identified clusters into T/natural killer cells, monocytes, epithelial cells, endothelial cells, fibroblasts, B cells, plasma cells, and dendritic cells (Figures 2B and 2C). Patient-specific cellular heterogeneity was evident in both proportion and abundance (Figure 2D). To quantify pyruvate metabolic activity in HCC, we integrated six orthogonal single-cell metabolic scoring algorithms through weighted averaging, referred to as “scoring.” Epithelial cells exhibited the highest scoring, followed by fibroblasts (Figure 2E). Tumor-derived epithelial cells demonstrated significantly elevated pyruvate metabolism scores compared to their regular tissue counterparts (Figure 2F), with cross-validation across five independent single-cell cohorts demonstrating a consistent metabolic disparity (Figures S1A–S1P). Building on these scores, we partitioned two distinct clusters of epithelial cells into two functionally distinct subpopulations: high-scoring (PyHighEpi) and low-scoring (PyLowEpi) clusters, to delineate metabolic heterogeneity in malignant progression. The integration of epithelial cells across all specimens enabled systematic comparative transcriptomics between tumor and paratumor tissues, identifying 25 differentially expressed pyruvate metabolism-related genes (DEPRGs) with differential expression patterns (Table S2).

### Pseudo-time analysis and intercellular communication analysis in scRNA

**Figure 3:**
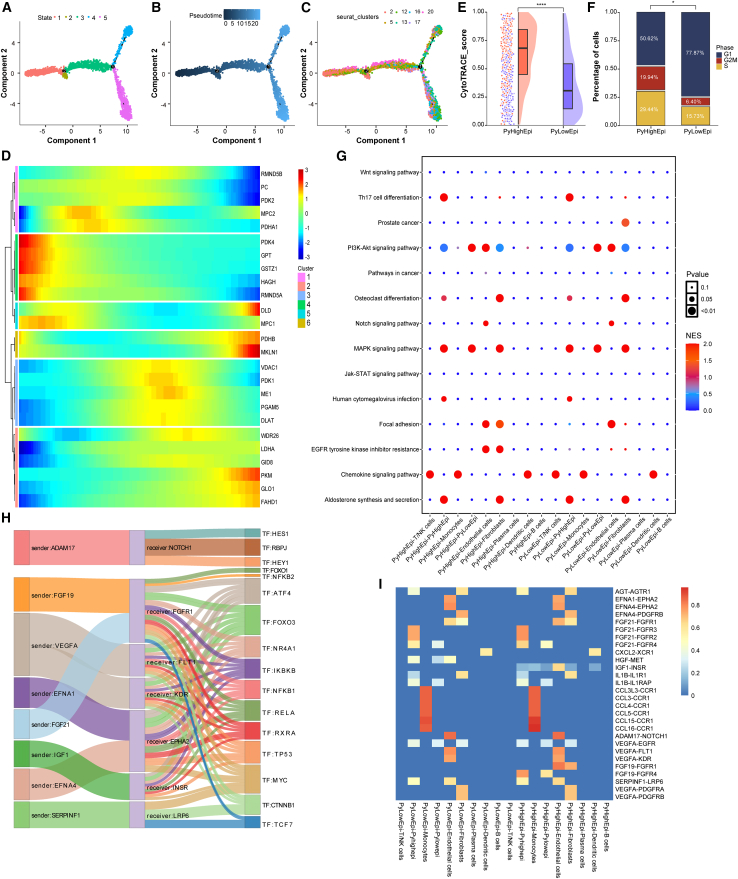
Pseudo-time analysis and intercellular communication in tumor epithelial cells in scRNA (A–C) Cell trajectory and pseudo-time analysis for the tumor epithelial cells. (D) Heatmap showing pseudo-time-dependent expression patterns of 25 pyruvate metabolism-related differentially expressed genes. (E) CytoTRACE scores distribution between PyHighEpi and PyLowEpi subclusters. (F) Cell-cycle phase distribution across PyHighEpi and PyLowEpi groups. (G) Bubble plots illustrate the activity analysis of signaling pathways in different cell types. (H) PyHighEpi and fibroblasts crosstalk: ligand receptor pairs and associated transcription factors. (I) Ligand receptor interactions between different cell types. ∗*p* < 0.05, ∗∗*p* < 0.01, ∗∗∗*p* < 0.001, ∗∗∗∗*p* < 0.0001, ns *p* > 0.05.

We determined cell trajectories and pseudo-time distributions of epithelial cells using the Monocle R package, revealing five distinct cellular states during malignant cell development, where states 4–5 represented terminal malignant progression (Figures 3A–3C). Temporal dynamics analysis of the 25 DEPRGs revealed stage-specific expression patterns: early-phase enrichment of PDK4, GPT, GSTZ1, HAGH, and RMND5A versus late-stage dominance of DLD, MKLN1, PKM, GLO1, and FAHD1 (Figure 3D). The PyHighEpi subpopulation demonstrated significantly higher CytoTRACE stemness than PyLowEpi subpopulations (Figure 3E). Furthermore, PyHighEpi cells displayed an increased proportion of cells in the S and G2M phases, indicating enhanced cell proliferation and accelerated progression (Figure 3F). Intercellular communication analyses using the CellCall tool revealed PyHighEpi-specific fibroblast intercellular crosstalk characterized by hyperactivation of the focal adhesion kinase (FAK) signaling and epidermal growth factor receptor (EGFR) tyrosine kinase inhibitor resistance pathway activity (Figure 3G). Notably, integrin β (ITGB) family members, key receptors in FAK signaling, are known to promote the expansion and self-renewal of cancer stem cells.^36^ Concurrent EGFR activation signatures corroborate prior reports linking this pathway to pyruvate-lactate metabolic reprogramming.^37^ Subsequently, we shifted our focus to potential TFs involved in the interaction between the two cell subpopulations. We found that NFKB1, NFKB2, MYC, and TP53 were activated, and all are associated with immune responses, cancer development, and/or metastasis (Figure 3H). Lastly, we examined potential ligand-receptor interactions between the PyHighEpi and PyLowEpi subclusters and their communication with other TME cell types (Figure 3I).

### ST reveals pyruvate-driven evolutionary trajectories in HCC

**Figure 4:**
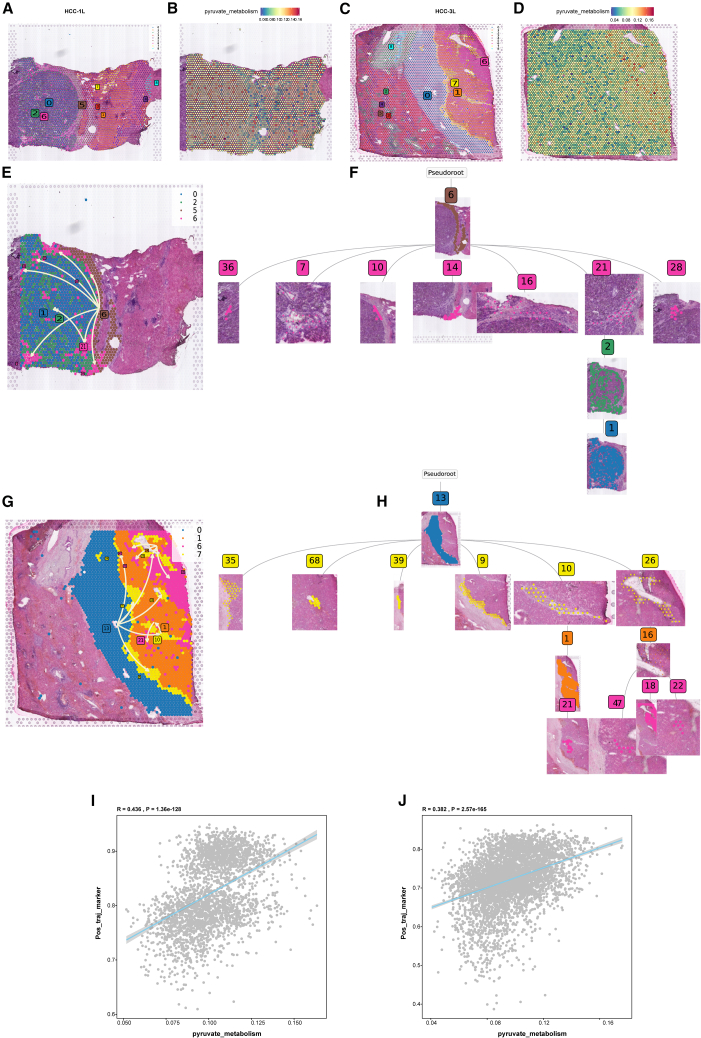
Spatial transcriptomics to identify pyruvate metabolism in HCC (A and C) Spatial distribution of 9 distinct clusters identified in HCC-1L and HCC-3L. (B and D) FeaturePlot of pyruvate metabolism in the spatial organization of HCC-1L and HCC-3L. (E and F) Spatiotemporal trajectory analysis of in HCC-1L. (G and H) Spatiotemporal trajectory analysis of in HCC-3L. (I and J) Correlation analysis between pyruvate metabolism genes and spatial trajectory scores in HCC-1L and HCC-3L.

Utilizing ST data from human HCC specimens (HRA000437, CNCB), we established a high-resolution cellular atlas through rigorous quality control and batch correction. Unsupervised clustering resolved nine histologically distinct cellular domains (Figures 4A and 4C). According to the original literature annotation, spatial mapping identified tumor core (HCC-1L: clusters 0/2/6; HCC-3L: clusters 1/6/7), stromal transition zones (HCC-1L: cluster 5; HCC-3L: cluster 0), and normal parenchyma.^38^ Notably, pyruvate metabolic scores showed a significant increase in tumor cores compared to adjacent normal tissue (Figures 4B and D). To decode spatiotemporal evolution patterns, we implemented pseudo-time trajectory reconstruction through stLearn’s diffusion-based algorithm. In HCC-1L, we hypothesized that the spatial trajectory originates from the stromal transition state (cluster 5) and evolves toward the tumor region (clusters 0, 2, and 6) (Figure 4E). The global spatial hierarchical dendrogram revealed that subclone 6 diverged into 7 evolutionary clades (36, 7, 10, 14, 16, 21, and 28), with the 21-2-1 branch representing the primary evolutionary trajectory, suggesting the presence of clonal competition within the tumor (Figure 4F). Similarly, HCC-3L progression originated from the stromal transition cluster (cluster 0) and propagated to tumor foci (clusters 1, 6, 7) (Figure 4G). Here, subclone 13’s dendrogram exhibited 6 distinct clades (35, 68, 39, 9, 10, and 26), with branch 10-1-21 marking the main evolutionary path (Figure 4H). Trajectory-aligned gene expression analysis identified genes positively (blue) or negatively (red) correlated with spatial progression (Figures S2A–S2F). We aggregated genes showing a positive correlation with spatial trajectories into a single gene set. Upon evaluating this gene set using scoring methods, we identified significant positive correlations between pyruvate metabolism scores and genes positively associated with spatial trajectories (HCC-1L: R = 0.436; HCC-3L: R = 0.328) (Figures 4I and 4J). This metabolic-spatial codependency suggests an evolutionary paradigm where pyruvate reprogramming at stromal interfaces fuels clonal expansion during malignant transformation.

### Integrative SMR and scRNA analyses identify FAHD1 as a pivotal regulator in HCC

**Figure 5:**
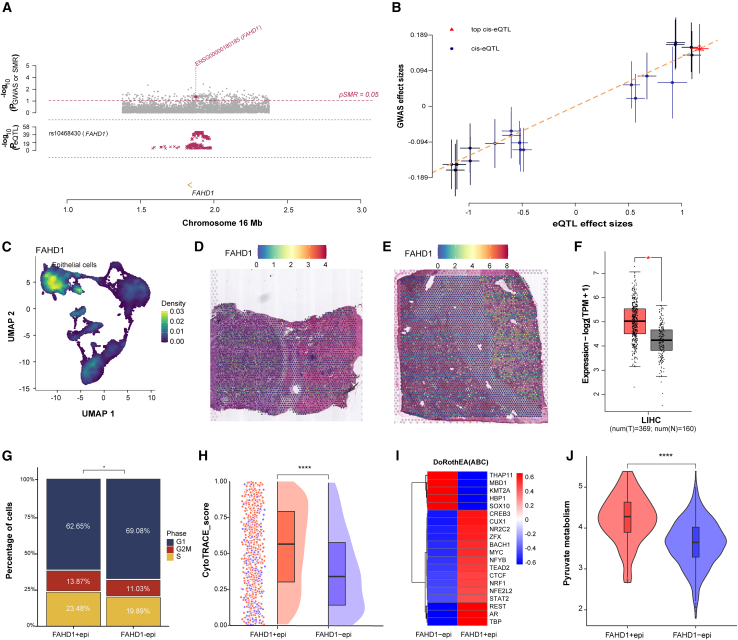
Summary data-based Mendelian randomization and scRNA analyses reveal FAHD1 in HCC (A) The summary data-based Mendelian randomization (SMR) locus plot. In the top plot, gray dots represent the *p* values for SNPs from the HCC GWASs; the diamond represents the *p* value for FAHD1 from the SMR test. The bottom plot shows the summary statistics of FAHD1. (B)The SMR effect plot. The horizontal axis represents the effect sizes of SNPs on FAHD1, while the vertical axis represents the effect sizes of SNPs on HCC risk. (C–F) FAHD1 expression across multiple datasets, including scRNA-seq, stRNA-seq (spatial transcriptome sequencing), and bulk RNA-seq (the GEPIA2 database (http://gepia2.cancer-pku.cn, using TCGA-LIHC and GTEx datasets). (G) Barplot showed cell-cycle states FAHD1+epi and FAHD1−epi groups. (H) Raincloud plot of CytoTRACE scores by FAHD1+epi and FAHD1−epi subclusters. (I) Heatmap showing transcriptional activity in FAHD1+epi and FAHD1−epi groups. (J) Violin plot showing pyruvate metabolism scores in FAHD1+epi and FAHD1−epi groups. ∗*p* < 0.05, ∗∗*p* < 0.01, ∗∗∗*p* < 0.001, ∗∗∗∗*p* < 0.0001, ns *p* > 0.05.

The SMR analysis employing rigorous IV selection identified FAHD1 as the sole DEPRGs, where genetic variants demonstrate a significant association with HCC susceptibility suggestive of a potential causal role (P_SMR = 0.02, P_HEIDI = 0.83, β = 0.129; Figures 5A and 5B; Table S3). Multi-omics validation across single-cell, spatial, and bulk transcriptomic datasets consistently confirmed FAHD1 overexpression in malignant cells and regions compared to their normal counterparts (Figures 5C–5F). Functional stratification of tumor epithelial cells into FAHD1+epi and FAHD1−epi subpopulations demonstrated that FAHD1+epi cells exhibited accelerated proliferative capacity with elevated S/G2M phase fractions, enhanced stem-like properties reflected by CytoTRACE scores, and augmented pyruvate metabolic activity (Figures 5G–5J). DoRothEA-based transcriptional network analysis further identified the activation of progression-associated TFs, including CREB3, MYC, STAT2, NFE2L2, and AR in FAHD1+epi cells (Figure 5I). These findings establish FAHD1 as a central molecular hub that integrates pyruvate metabolic reprogramming with transcriptional regulation during HCC malignant evolution.

### Spatial transcriptome analyses uncover FAHD1 in HCC

**Figure 6:**
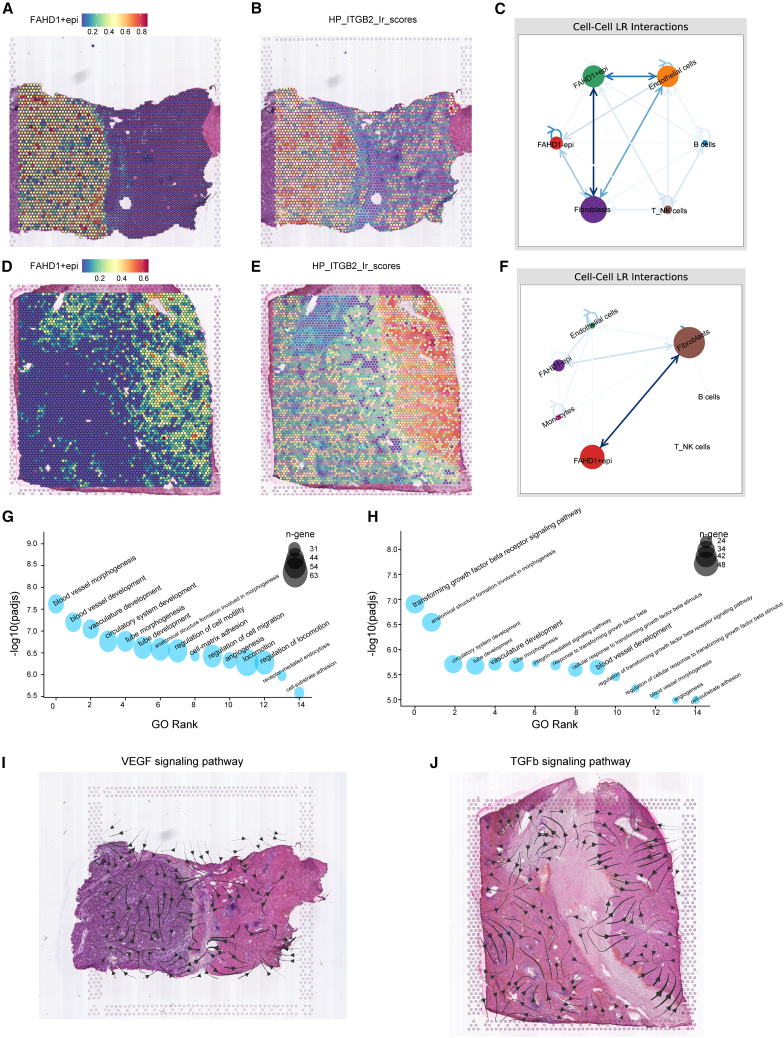
Spatial transcriptomic profiling reveals FAHD1-associated signaling networks in HCC (A and D) Spatial distribution of FAHD1+epi cells in HCC-1L and HCC-3L (RCTD-based analysis). (B and E) The spatial plot of HP-ITGB2 ligand-receptor pair activity scores in HCC-1L and HCC-3L. (C and F) Cell-cell communication networks across distinct cell populations in the spatial context of HCC-1L and HCC-3L. (G and H) Gene Ontology enrichment of top-ranked ligand-receptor pairs in HCC-1L and HCC-3L. (I and J) Spatial trajectory analysis of VEGF and TGF-β pathway signaling in HCC-1L and HCC-3L.

To gain a deeper understanding of the role of FAHD1 in HCC, we conducted an in-depth analysis of spatial transcriptome analyses. Subsequently, we applied the RCTD methodology, juxtaposing spatially resolved transcriptomic data with scRNA-seq data. The results showed that tumor regions in HCC-1L and HCC-3L showed predominant localization of FAHD1+epi (Figures 6A and 6D). Intercellular communication analysis via stLearn revealed enhanced ligand-receptor interactions between FAHD1+epi and fibroblasts compared to other epithelial subsets (Figures 6C and 6F). The top-ranked interaction pair, HP_ITGB2, was enriched explicitly in FAHD1+epi regions (Figures 6B and 6E). Notably, ITGB2 has been implicated in cancer-associated fibroblast (CAF)-mediated glycolytic activation and pyruvate-lactate secretion,^39^ suggesting a feedforward loop where FAHD1+epi cells engage CAFs through ITGB2 to reinforce metabolic symbiosis. Functional enrichment analysis highlighted that these prominent ligand-receptor pairs were mainly associated with vascular development and the transforming growth factor β (TGF-β) signaling pathway (Figures 6G and 6H). Directional signaling flux analysis (COMMOT algorithm) further demonstrated the spatial polarization of vascular endothelial growth factor (VEGF) and TGF-β pathways: VEGF signals accumulated within the tumor regions of HCC-1L, whereas TGF-β signals originated from stromal compartments and converged onto the tumor regions in HCC-3L (Figures 6I and 6J). These spatial patterns suggest microenvironmental crosstalk where FAHD1+epi cells engage CAF through ITGB2-mediated metabolic coupling and TGF-β/VEGF-driven niche remodeling.

### Development and validation of an FAHD1-driven prognostic signature

**Figure 7:**
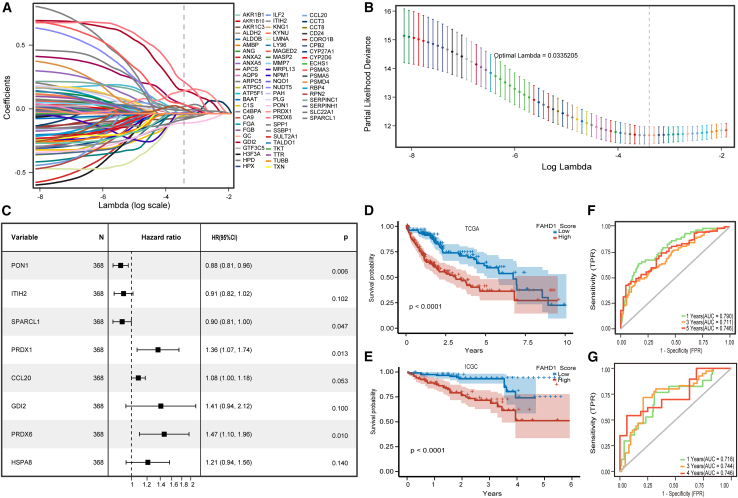
Development and validation of FAHD1-derived risk score prognostic signature for HCC prognosis (A) Lasso coefficient profiles. (B) Cross-validation is used to tune parameter selection in the Lasso model. (C) Forest plot of the 8 genes selected for the final prognostic model based on multiple Cox regression analysis. (D and E) Kaplan-Meier survival curves comparing high and low FAHD1-derived risk score (FRS) groups in TCGA (training) and ICGC (validation) cohorts. (F and G) Time-dependent ROC curves show the predictive performance of the FRS for overall survival in TCGA and ICGC cohorts.

To establish an FAHD1+epi-derived prognostic signature, we performed stepwise feature selection on scRNA-seq data. Initial marker gene screening (FindMarkers) identified 235 genes associated with FAHD1+epi (Table S4). Univariate Cox regression analysis revealed 90 genes significantly correlated with OS (Figure S3A; Table S5). Subsequent Lasso-Cox regularization refined the signature to 17 candidate genes (Figures 7A and 7B; Table S6). Next, we scrutinized these 17 genes using multiple Cox regression analyses and finally selected 8 genes to build a prognostic model (Figure 7C), which was used to calculate the FRS for each patient. The FRS demonstrated robust prognostic stratification in both the discovery and validation cohorts, with patients exhibiting a high FRS showing significantly reduced OS (Figures 7D and 7E). Moreover, the receiver operating characteristic (ROC) curves revealed the robust performance of the FRS in predicting OS in both cohorts (Figures 7F and 7G). Univariate and multivariate Cox regression, adjusted for clinical covariates (age, gender, and TNM stage), established FRS as an independent prognostic indicator (Figures S4A and S4B). Subgroup analyses revealed consistent prognostic performance across TNM stages and age groups, although sex-specific stratification was not observed in female patients (Figures S4C–S4H).

### Immunotherapy prediction, drug prediction, immune landscape, and molecular docking analyses

**Figure 8:**
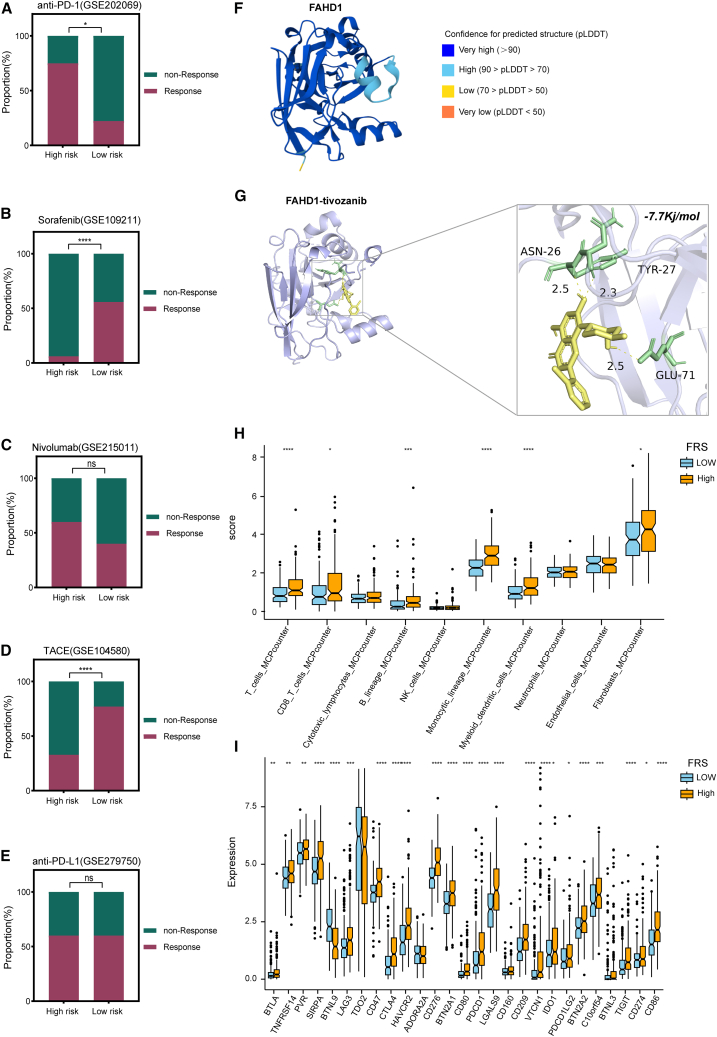
Immunotherapy prediction, drug prediction, Immune landscape, and molecular docking analyses (A–E) Predictive analysis of high-risk patients’ responses to different first-line therapies, including TACE, sorafenib, nivolumab, anti-PD-1, and anti-PD-L1. (F) A structural model of FAHD1 was generated using AlphaFold2. (G) Illustration of the FAHD1-tivozanib binding interaction, with a close-up view highlighting local docking details. (H and I) Differences in immune infiltration and immune checkPOINT expression across different FRS subgroups. ∗*p* < 0.05, ∗∗*p* < 0.01, ∗∗∗*p* < 0.001, ∗∗∗∗*p* < 0.0001, ns *p* > 0.05.

To evaluate the therapeutic response in high-risk patients, cohorts treated with TACE, sorafenib, nivolumab, anti-PD1, and anti-PD-L1 were selected for predictive analysis. High-risk patients exhibited a greater response to nivolumab and anti-PD1 therapy (Figures 8A and 8C). While some responded positively to sorafenib, TACE, and anti-PD-L1, overall efficacy remained suboptimal (Figures 8B, 8D, and 8E). These findings indicate that conventional first-line treatments may be insufficient for high-risk patients, highlighting the need for novel therapeutic strategies. To identify potential drug candidates with increased efficacy in this subgroup, the CMap tool was utilized. This analysis yielded top-10 promising compound candidates predicted to reverse pathological gene expression patterns, including tivozanib, RO-3306, and triptolide, and others (Table S7). To evaluate the binding potential between FAHD1 and tivozanib, a molecular docking study was performed. Five FAHD1 models were generated using AlphaFold2 based on the FASTA sequence (Table S8), with the top-ranked model achieving a predicted local distance difference test (pLDDT) score of 93 (Figure 8F; Table S9). Molecular docking conducted using AutoDock Vina version 1.5.7 yielded a binding energy of −7.7 kcal/mol for the FAHD1-tivozanib complex, suggesting a highly stable interaction (Figure 8G). Immune infiltration analysis revealed distinct characteristics of the high-FRS groups, including significantly enhanced immune cell infiltration, particularly in T cells, CD8^+^ T lymphocytes, and monocytic lineages, along with marked upregulation of key immune checkpoint molecules such as PD-1 and CTLA4 (Figures 8H and 8I). This suggests that despite enhanced immune cell infiltration, immune tolerance and escape may occur within the TME, leading to T cell function depletion, which may explain why our high-risk patients were more sensitive to anti-PD-1 therapy.

### Experimental validation of FAHD1

**Figure 9:**
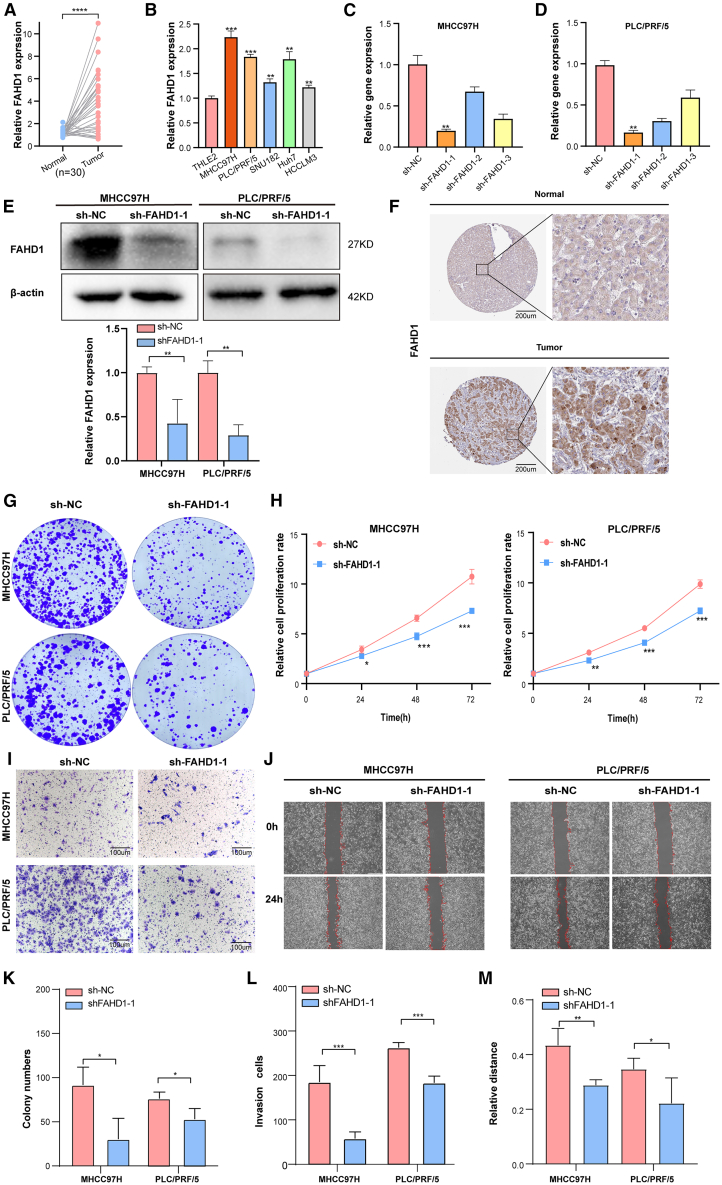
Role of FAHD1 in HCC (A and B) Quantitative real-time PCR analysis of FAHD1 expression in 30 paired HCC and adjacent normal tissues, as well as in normal liver cells (THLE2) and five HCC cell lines (MHCC97H, PLC/PRF/5, SNU-387, Huh7, and HCCLM3). (C and D) Quantitative real-time PCR validation of FAHD1 silencing in MHCC97H and PLC/PRF/5 cells transfected with shRNA targeting FAHD1 (sh-FAHD1-1, sh-FAHD1-2, and sh-FAHD1-3). (E) Western blot analysis confirmed effective FAHD1 knockdown in MHCC97H and PLC/PRF/5 cells, with β-actin as a loading control. The lower panels show the quantification of FAHD1 protein levels. (F) Immunohistochemical analysis of FAHD1 on HCC tissue and normal tissue. Scale bar, 200 μm. (G and K) Colony-formation assay in MHCC97H and PLC/PRF/5 cells with sh-NC and sh-FAHD1-1. (H) Cell viability was detected using CCK-8 assays. (I and L) Transwell invasion assay in MHCC97H and PLC/PRF/5 cells. Scale bar, 100 μm. (J, M, and N) Wound-healing assay in MHCC97H and PLC/PRF/5 cells at 0 and 24 h. ∗*p* < 0.05, ∗∗*p* < 0.01, ∗∗∗*p* < 0.0001, ∗∗∗∗*p* < 0.001, ns *p* > 0.05. Error bars represent mean ± SD (*n* = 3).

To investigate the role of FAHD1 in HCC, we systematically assessed its expression and functional impact across clinical specimens and cellular models. Analysis of 30 paired HCC tumor and adjacent normal tissues revealed significant upregulation of FAHD1 mRNA levels in tumors (Figure 9A). To exclude cell line-specific artifacts, we validated FAHD1 overexpression across five independent HCC cell lines (MHCC97H, PLC/PRF/5, SNU182, Huh7, and HCCLM3) compared to normal human hepatocytes (THLE-2). FAHD1 was significantly upregulated in all HCC cell lines, with MHCC97H and PLC/PRF/5 exhibiting the most pronounced expression levels (Figure 9B). Therefore, they were selected for subsequent functional studies. To elucidate the mechanistic role of FAHD1 in HCC progression, we designed three independent shRNA constructs (sh-FAHD1-1, -2, -3) for gene silencing to minimize off-target effects. Quantitative real-time PCR and western blot analyses demonstrated that sh-FAHD1-1 achieved the highest knockdown efficiency (Figures 9D and 9E) and was consequently employed for all subsequent functional assays. Immunohistochemistry analysis from the Human Protein Atlas (HPA) database (https://www.proteinatlas.org, antibody HPA043226) further supported these findings, showing significantly higher FAHD1^+^ staining in tumor tissues than in normal tissues (Figure 9F). Functionally, FAHD1 knockdown led to a significant reduction in colony formation in the sh-FAHD1-1 group compared to the sh-NC (negative control) group (Figure 9G). Longitudinal CCK-8 assays demonstrated a marked decrease in cell growth in both HCC cell lines following FAHD1 knockdown at all assessed time points (Figure 9H). Furthermore, transwell invasion assays revealed that FAHD1 silencing significantly impaired the invasive capacity of MHCC97H and PLC/PRF/5 cells (Figures 9I and 9L). Consistently, wound-healing assays demonstrated a substantial reduction in migratory ability upon FAHD1 knockdown (Figures 9J–9M). Collectively, these findings establish FAHD1 as a key driver of HCC progression, promoting tumor cell proliferation, migration, and invasion.

## Discussion

Pyruvate, a key metabolic intermediate linking glycolysis and mitochondrial oxidation, plays a critical role in tumor biology.^40^ In HCC, pyruvate flux is frequently skewed toward aerobic glycolysis (the Warburg effect), bypassing OXPHOS to meet the bioenergetic and biosynthetic demands of proliferating cells.^41^^,^^42^^,^^43^ Beyond fueling anabolic growth, this metabolic shift actively shapes the TME by promoting immunosuppression, angiogenesis, and extracellular matrix (ECM) remodeling—processes that collectively drive tumor progression and resistance.^44^^,^^45^^,^^46^^,^^47^ Therapeutic targeting of pyruvate metabolism, exemplified by pyruvate dehydrogenase (PDH) kinase (PDK) inhibitors such as dichloroacetate (DCA), has shown promise in preclinical models.^48^^,^^49^^,^^50^ However, clinical translation remains limited, with paradoxical reports of DCA exacerbating tumor progression in specific contexts.^51^^,^^52^ These challenges underscore the need to identify context-specific regulators of pyruvate metabolism that govern HCC progression.

In this study, we systematically examined the role of pyruvate metabolism in the TME of HCC, utilizing single-cell transcriptomics, spatial mapping, and causal genetic inference approaches. Our findings reveal that pyruvate metabolism is upregulated in tumor epithelial cells, as assessed through various scoring methods. Specifically, PyHighEpi cells exhibited enhanced stemness and metastatic potential, highlighting pyruvate metabolism as a critical determinant of tumor aggressiveness and therapeutic resistance. MYC, a key regulator of oncogenic metabolism, promotes glycolysis (via LDHA, PFK1), glutamine metabolism (via GLS1), and mitochondrial biogenesis to fuel rapid tumor proliferation.^53^^,^^54^ In PyHighEpi cells, MYC-driven metabolic reprogramming likely sustains hyperactivated pyruvate metabolism while concurrently inducing stemness-associated TFs, such as SOX2 and KLF4, which reinforces their dedifferentiated phenotype and metastatic potential.^55^ To further investigate the relationship between pyruvate metabolism and tumor progression, we initiated a comprehensive study of the spatial transcriptome. We observed an elevated pyruvate metabolic signature score in the core of HCC tumors, a result consistent with our scRNA data. Using stlearn, we mapped the evolutionary trajectories of HCC and found a positive correlation between the transition of evolutionary genes from stromal to tumor regions and the metabolism of pyruvate. This suggests that increased pyruvate metabolism within tumors is associated with a greater tendency for tumor progression, reinforcing our assertion that pyruvate metabolism catalyzes the malignant evolution of HCC. To dissect the molecular drivers of pyruvate metabolism in HCC, we performed SMR, which identified FAHD1 as a causal risk gene for HCC. Following the existing literature, FAHD1 emerges as a mitochondrial enzymatic regulator that orchestrates metabolic reprogramming by catalyzing the decarboxylation of oxaloacetate (OAA) and modulating the activity of succinate dehydrogenase (SDH/complex II), thereby reshaping cellular metabolism.^56^ Through PC suppression-mediated restriction of OAA regeneration,^57^ FAHD1 constrains TCA cycle progression while destabilizing the phosphoenolpyruvate (PEP)-pyruvate-OAA metabolic axis, which is essential for maintaining metabolic plasticity in proliferating tumor cells.^58^^,^^59^ FAHD1-driven OAA decarboxylation depletes mitochondrial guanosine diphosphate, a key modulator of SDH activity, leading to the suppression of the electron transport chain (ETC).^60^^,^^61^ This inhibition potentially reduces electron leakage-mediated ROS generation, conferring metabolic homeostasis that enhances neoplastic cell adaptation to oxidative stress.^59^ This regulatory mechanism differs from the conventional pyruvate dehydrogenase (PDH)-PDK axis, suggesting that FAHD1, as a crucial regulator of tumor metabolic reprogramming, may serve as a potential target for cancer therapy and metabolic interventions.

Beyond its tumor-intrinsic effects, we observed FAHD1+epi cells actively reshaping the TME through ITGB2-dependent crosstalk with CAFs. It is well established that CAFs promote tumor growth, angiogenesis, invasion, metastasis, and chemotherapy resistance through various mechanisms, including ECM remodeling.^62^^,^^63^ Notably, studies have confirmed the metabolic coupling between CAFs and tumor cells.^64^ Our ligand-receptor interaction analysis revealed strong enrichment of TGF-β and VEGF signaling, suggesting that FAHD1+epi cells establish a metabolic niche that not only supports vascular abnormality but also exacerbates immune evasion. TGF-β is known to suppress antitumor immunity by inducing regulatory T cells and inhibiting the infiltration of cytotoxic T lymphocytes. In contrast, VEGF-mediated vascular remodeling promotes immune exclusion by impairing the trafficking of antigen-presenting cells and T cell extravasation.^65^^,^^66^ These findings indicate that FAHD1 may contribute to immunotherapy resistance by shaping an immunosuppressive TME, warranting further investigation into FAHD1-targeted strategies to overcome immunotherapy resistance in HCC. Clinically, the FRS demonstrated substantial prognostic value, effectively stratifying patients based on their survival outcomes and responsiveness to immunotherapy. CMap analysis identified tivozanib, a selective VEGFR-1/-2/-3 inhibitor with established antiangiogenic efficacy in renal cell carcinoma,^67^ as the top candidate for reversing pathological gene signatures in high-risk HCC. Notably, molecular docking analysis revealed a high-affinity interaction between tivozanib and FAHD1 (binding energy −7.7 kcal/mol), reinforcing our findings and highlighting a potential avenue for targeting pyruvate metabolism.

Despite these advances, key questions remain. First, the precise biochemical interplay between FAHD1, SDH, and ETC components requires validation using isotopic flux analysis and mitochondrial proteomics. Second, the ITGB2-CAF axis, although computationally inferred, requires functional interrogation in three-dimensional co-culture systems to distinguish between metabolic and mechanical cross-talk. Third, although mechanistic studies support the integrated OAA tautomerase and decarboxylase activities of FAHD1, wherein tautomerism facilitates efficient decarboxylation to yield pyruvate and mitigates enol-OAA-mediated SDH inhibition, their roles in inhibiting PC and enabling the PEP-pyruvate-OAA axis in HCC cells require confirmation through direct flux measurements and enzyme kinetics assays. The prospective validation of FRS in immunotherapy-treated cohorts is crucial for assessing its utility in guiding therapeutic stratification. Lastly, while our molecular docking analysis indicates tivozanib as a potential FAHD1 inhibitor, we acknowledge that this is preliminary and lacks direct validation in HCC cell lines. Future studies will focus on targeted enzymatic and cellular assays to confirm these interactions and evaluate the potential for therapeutic repurposing.

### Conclusion

Our study repositions pyruvate metabolism from a hallmark of cancer metabolism to a spatial organizer of HCC evolution. By identifying FAHD1 as a linchpin connecting mitochondrial reprogramming, stromal co-option, and immune evasion, we provide a roadmap for targeting metabolic plasticity to disrupt tumor ecosystems. Future efforts to therapeutically exploit this axis may benefit from dual-pronged strategies that simultaneously cripple tumor-intrinsic metabolism (e.g., FAHD1 inhibition) and remodel the immunosuppressive niche, potentially overcoming limitations observed with earlier metabolic agents like DCA.

## Data and code availability

The scRNA-seq data generated during this study are available at the GEO under accession numbers GSE149614, GSE125449, GSE189903, GSE202642, GSE151530, GSE290925 (https://www.ncbi.nlm.nih.gov/geo/). ST data are available from the CNCB (https://www.cncb.ac.cn/). Bulk transcriptomics data are available at TCGA-LIHC (https://portal.gdc.cancer.gov/) and ICGC (https://dcc.icgc.org/). Pyruvate metabolism gene sets are available at the GSEA database (https://www.gsea-msigdb.org/gsea/index.jsp). SMR analysis utilized liver tissue *cis*-eQTL data from the Yang lab (https://yanglab.westlake.edu.cn/software/smr/#Overview), and GWAS data can be accessed from the FinnGen_R8 cohort (https://www.finngen.fi/en/access_results).

## Acknowledgments

We are grateful for the publicly available databases and the efforts of those who maintain them, as well as Bioicons (https://bioicons.com/) for providing the vector graphics used in this study. This study was supported by the Joint Project on Regional High-Incidence Diseases Research of Guangxi Natural Science Foundation under grant nos. 2024GXNSFBA010049 and 2023GXNSFDA026001 and the 10.13039/501100001809National Natural Science Foundation of China (grant no. 82260419).

## Author contributions

J.H., writing – original draft, visualization, and data curation; S.L., investigation and formal analysis; J.S., writing – original draft and methodology; H.C., writing – review & editing, funding acquisition, and conceptualization.

## Declaration of interests

The authors declare no competing interests.

## Supplemental information

Data S1. Tables S1–S9

Document S2. Article plus supplemental information
