# Supplementary material for: FAHD1-mediated pyruvate metabolism in hepatocellular carcinoma: Multi-omics and causal genetic evidence
Source: HGG Adv. 2025 Aug 14;6(4):100494. doi: 10.1016/j.xhgg.2025.100494 (PMC12414894; doi:10.1016/j.xhgg.2025.100494)
Supplement: Document S1. Figures S1–S4 [file mmc1.pdf]

HGGA, Volume *6*

Supplemental information

FAHD1-mediated pyruvate metabolism in hepatocellular carcinoma: Multi-omics and causal genetic evidence

Jin Huang, Shijie Liang, Jiamin Sun, and Huaping Chen

Supplementary figures

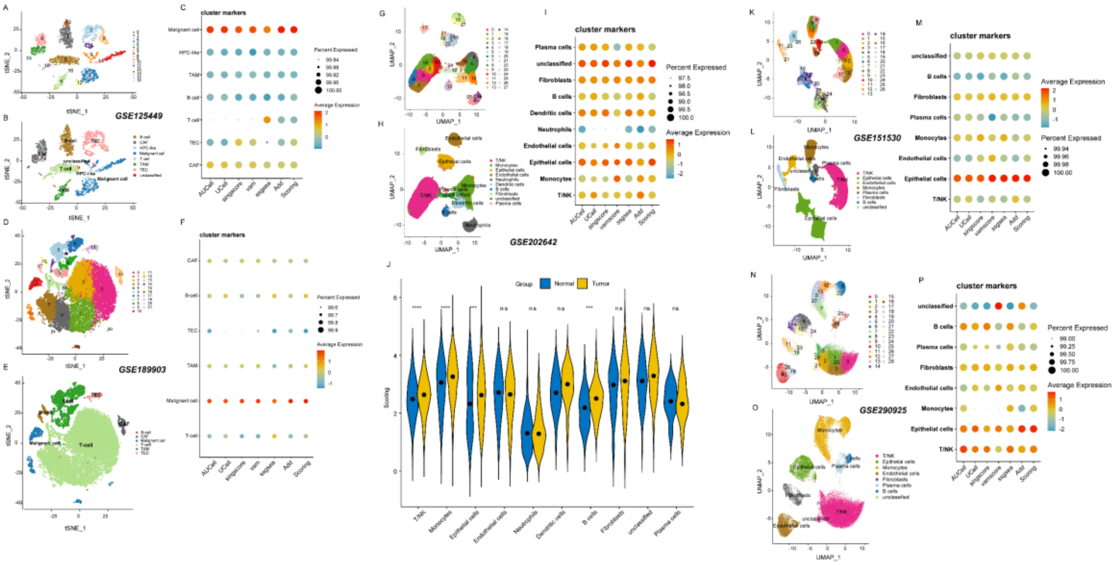

Supplementary Fig. 1: Pyruvate metabolism is increased in malignant cells and is confirmed in independent single-cell datasets:**(A)** T-SNE analysis divided 3,913 cells into 16 cell clusters. **(B)** These 16 clusters were classified into eight cell types. **(C)** Bubble plot depicting multi-method pyruvate metabolism scores across different cell types in GSE125449. **(D)** T-SNE analysis identified 21 cell clusters from 25,907 cells. **(E)** These 21 clusters were classified into six cell types. **(F)** Bubble plot depicting multi-method pyruvate metabolism scores across different cell types in GSE189903. **(G)** UMAP analysis identified 28 cell clusters from 90,247 cells. **(H)** These 28 clusters were classified into 10 cell types. **(I)** Bubble plot depicting multi-method pyruvate metabolism scores across different cell types in GSE202642. **(J)** Violin plot illustrating metabolic scores stratified by tissue type. **(K)** UMAP analysis identified 27 cell clusters from 45,769 cells. **(L)** These 27 clusters were classified into eight cell types. **(M)** Bubble plot depicting multi-method pyruvate metabolism scores across different cell types in GSE151530. **(N)** UMAP analysis identified 29 cell clusters from 125,518 cells. **(O)** These 29 clusters were classified into eight cell types. **(P)** Bubble plot depicting multi-method pyruvate metabolism scores across different cell types in GSE290925.

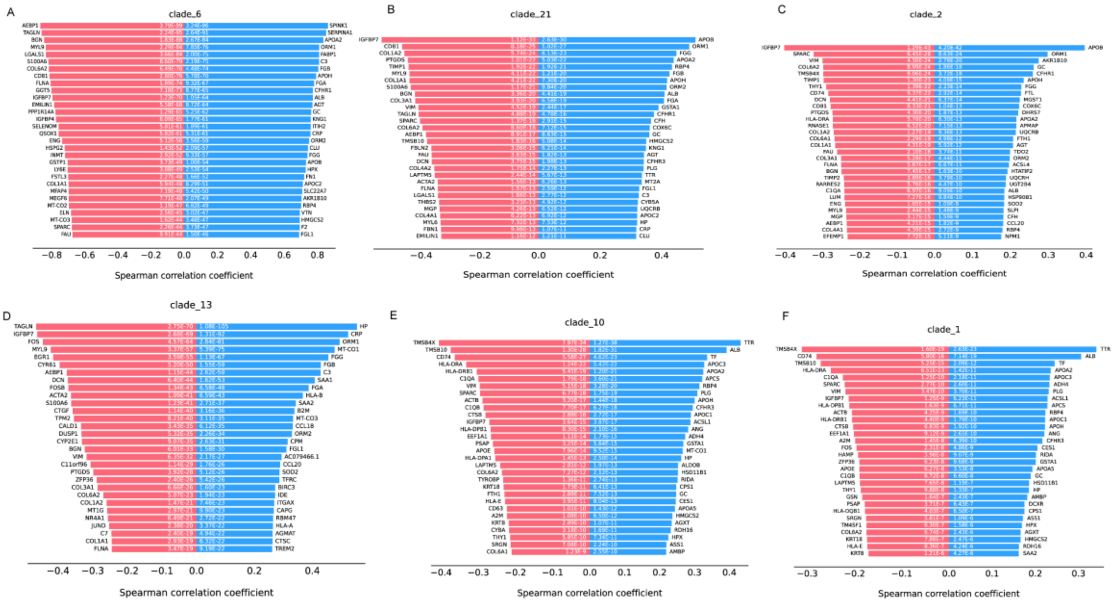

Supplementary Fig. 2: Spatial trajectory genes in HCC-1L and HCC-3L: red for negative, blue for positive: **(A-C)** Spatial trajectory genes in HCC-1L. **(D-E)** Spatial trajectory genes in HCC-3L.

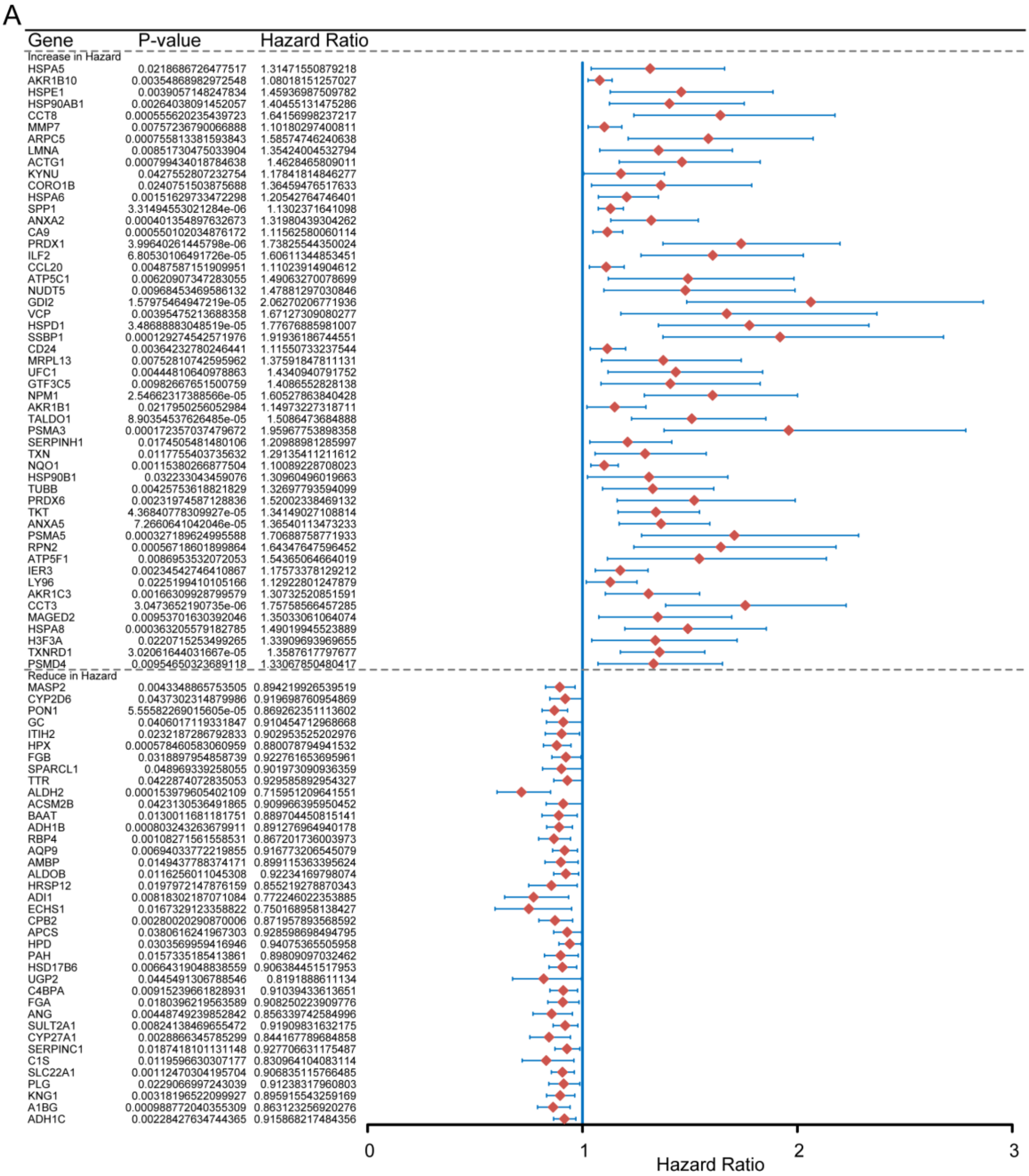

Supplementary Fig. 3: Univariate Cox regression analysis: **(A)** 90 genes were screened using Univariate Cox regression analysis.

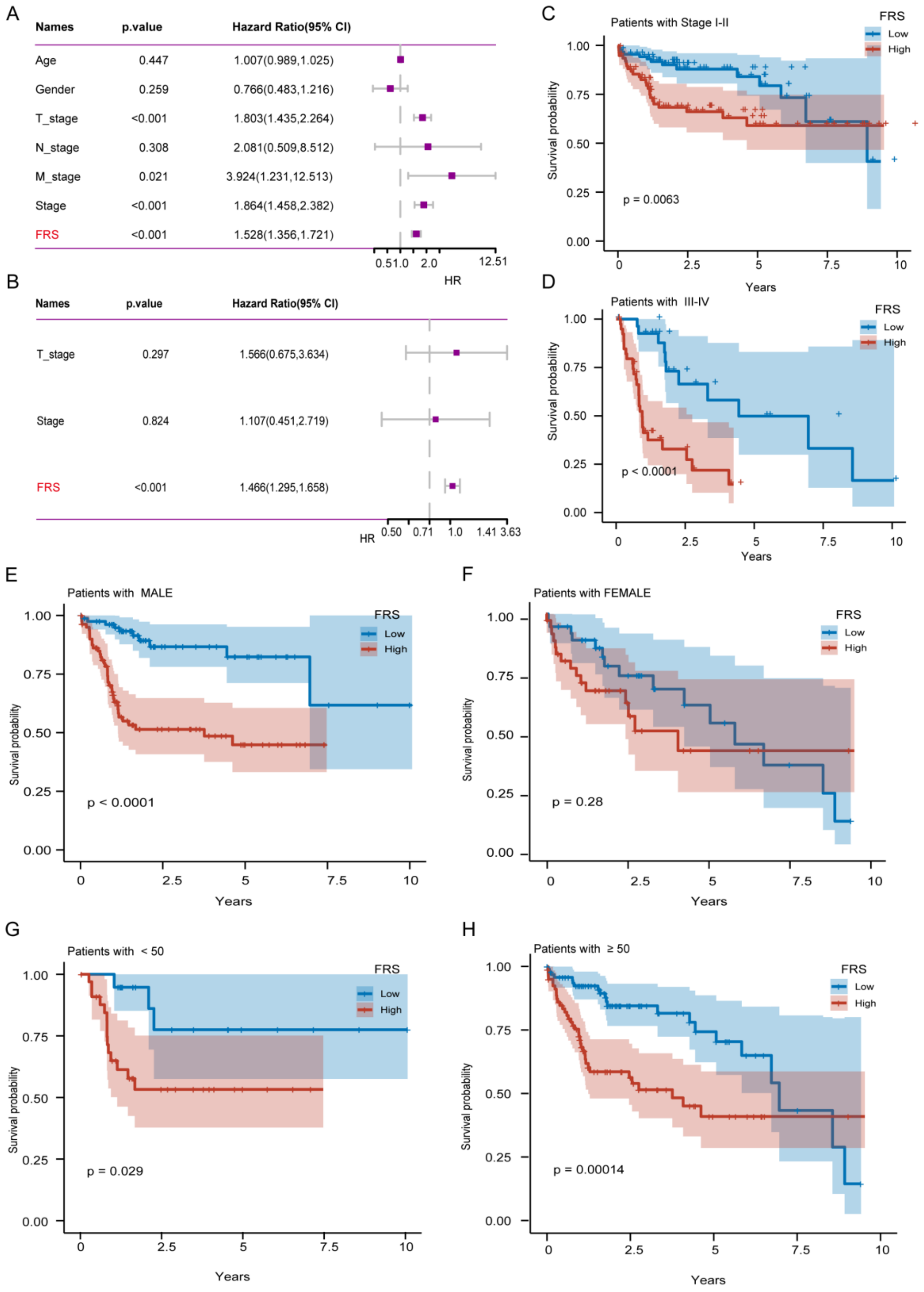

Supplementary Fig. 4: FRS can serve as an independent prognostic factor for HCC: **(A)** Forest plot of univariate Cox regression analysis for FRS and clinical characteristics in TCGA cohort. **(B)** Forest plot of multivariate Cox regression analysis confirming FRS as an independent prognostic factor. **(C-H)** Kaplan-Meier survival curves of subgroup analyses based on age, gender, and stage.
